# Study of the Relationship between Leaf Color Formation and Anthocyanin Metabolism among Different Purple Pakchoi Lines

**DOI:** 10.3390/molecules25204809

**Published:** 2020-10-19

**Authors:** Bo Song, Hai Xu, Longzheng Chen, Xiaoxue Fan, Zange Jing, Song Chen, Zhigang Xu

**Affiliations:** 1 Key Laboratory of Southern Vegetable Crop Genetic Improvement in Ministry of Agriculture College of Horticulture, Nanjing Agricultural University, Nanjing 210095, China; anybody119@sina.com (B.S.); 2016201010@njau.edu.cn (S.C.); 2 Jiangsu Key Laboratory for Horticultural Crop Genetic Improvement, Institute of Vegetable Crops, Jiangsu Academy of Agricultural Sciences, Nanjing 210014, China; xuhai407@163.com (H.X.); Chenglong4510@sohu.com (L.C.); fxx600@163.com (X.F.); 3 College of Agriculture and Life Science, Kunming University, Kunming 650214, China; Jingzange@aliyun.com

**Keywords:** pakchoi, purple leaf, color formation, anthocyanin

## Abstract

Purple pakchoi (*Brassica rapa* ssp. *Chinensis*) is particularly appreciated due to its high edible quality and ornamental value, but there are few studies on the underlying mechanisms of leaf color formation. To comprehensively assess the differences in purple formation in pakchoi, four lines of pakchoi with different purple leaves were used in this experiment to determine the pigment content and to investigate the distribution and components of anthocyanin using LCMS (Liquid Chromatography Mass Spectrometry) and leaf cross-sections. Moreover, the expression levels of anthocyanin synthesis-related genes in four lines were calculated by qRT-PCR. The results showed that three new purple lines rich in anthocyanin and of high-quality were bred, and the anthocyanin were mainly distributed in both the upper epidermis and lower epidermis of leaves. Thirteen anthocyanin components were separated and identified, all the anthocyanins were acylated and glycosylated cyanidins; the main anthocyanins in purple pakchoi were a diacylated form of cyanidin 3-*trans*-(feruloyl)diglucoside-5-(malonyl)glucoside. Both the ratio of non-aromatic acylated cyanidin to aromatic acylated cyanidin and the ratio of anthocyanin content to chlorophyll content were responsible for the color formation in different purple pakchoi lines. When the ratio was high, the leaf appeared reddish purple, and when the ratio was low, the leaf appeared deep purple, even blackish purple. The expression level of *BrF3H* was significantly correlated with the content of anthocyanin through the correlation coefficient, which was speculated to be the main anthocyanin synthesis-related gene resulting in color differences among the four purple pakchoi lines. These results will enhance our understanding for the cultivation of new purple pakchoi varieties.

## 1. Introduction

Pakchoi (*Brassica rapa* ssp. *Chinensis*), which is native to China, is increasingly popular due to its high nutritional value, rapid growth and wide adaptability. Today, pakchoi has been widely introduced in Southeast Asia, Japan, the United States and European countries [1,2]. It is not only suitable for eating, but also for ornamental applications [3]. For example, the leaf color among pakchoi cultivars range from green and yellow to deep purple; in particular, new purple lines (e.g., blackish purple, reddish purple, deep reddish purple) have been successfully created by our research team in recent years, enriching the ornamental germplasm resources of pakchoi. However, little is known about the mechanisms regulating the leaf color formation of purple pakchoi.

The color formation of the leaves was attributed to the comprehensive effect of pigments such as chlorophyll, anthocyanin and carotenoid [4]. Previous studies showed that the change in the ratio of anthocyanin content to chlorophyll content affected the change in the color of plants with colorful leaves such as Kotinus [5], Spathe [6] and Red Maple [7]. Meanwhile, while the tissue structure of the leaf led to the different colored phenotypes, a previous report showed that anthocyanin existed only in the upper epidermis in the deep purple pakchoi line [8,9]. However, the content and distribution of pigments underlying leaf color in new purple pakchoi lines are poorly understood.

Many plants with colorful leaves have cultivars with distinct leaf colors; the variety of leaf colors is mainly attributed to the chemical structures of different anthocyanins accumulated in the leaf [10]. The anthocyanins in plants, including cyanidin, delphinidin, pelargonidin, peonidin, petunidin and malvidin, cause multiple colors, including blue, yellow, red and orange [11,12]. In *Brassica* vegetables, fifteen to twenty-four anthocyanins from the leaves of red cabbage were detected by HPLC [13,14,15]. Twenty to thirty kinds of anthocyanins have been identified in purple Chinese cabbage [16,17,18]. In the deep purple pakchoi line, eight to twenty species of anthocyanins have been investigated [16,19,20]. Among them, acylated cyanidin glycosides are the main accumulated anthocyanin components. However, the composition of anthocyanins in the leaves of purple pakchoi have not been investigated in relation to leaf color. Recently, information on anthocyanin biosynthesis genes in pakchoi has been sparse. Research has shown that anthocyanin synthesis-related genes (*PAL*, *CHS*, *F3H*, *DFR*, *ANS* and *UFGT*) in purple pakchoi were expressed significantly more highly than that of green pakchoi by transcriptome sequencing [21], but there has been no further study on the relationship between the expression patterns of anthocyanin biosynthetic genes and pigment accumulation in the diverse leaf color of purple pakchoi lines.

In this study, we firstly observed agronomic characters and anatomical structures, and determined the pigment content in relation to four pakchoi lines with different purple colors. Next, we performed an analysis of the anthocyanin components, and investigated the relationship between the expression patterns of anthocyanin synthesis-related genes and anthocyanin accumulation. These results could clarify the mechanism regulating the leaf color formation of purple pakchoi.

## 2. Results and Discussion

### 2.1. Measurement of Agronomic Characters

The value of agronomic characters in four purple pakchoi lines were presented in Table 1. Except the “PQC” (Purple Qing Cai) line, the front and back of the leaf in the lines of “PHXW” (Purple Huang Xin Wu) “RWTC” (Red Wu Ta Cai) and “RSH” (Red Shang Hua) were well colored. Significant differences in eight major agronomic traits were found in four lines, indicating that large genetic differences existed among lines with different purple leaves. All trait values of the “PHXW” line were the smallest, with a weight of 160.00 g per plant, while those of the “RWTC” line and “RSH” line were larger, with a weight of 596.67 g and 415.00 g per plant, respectively.

### 2.2. Anatomy Observations

**Figure 1:**
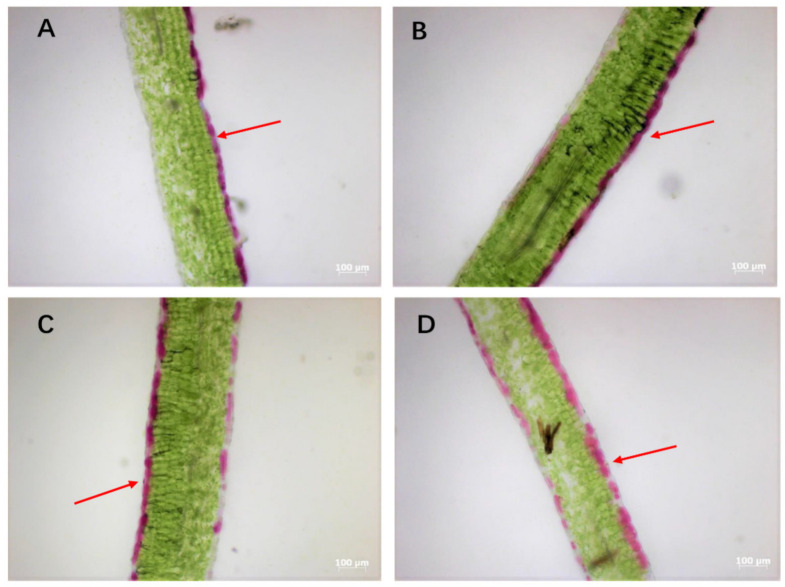
Anatomical observation of leaves from four purple pakchoi lines. Samples **A**, **B**, **C**, **D** are from the lines of “PQC”, “PHXW”, “RWTC” and “RSH”, respectively. The scale bar is 100 μm in **A**–**D**, respectively. Arrow heads indicate anthocyanin accumulation in the upper epidermis.

As shown in Figure 1, the anatomical structures of the leaves among four purple pakchoi lines were observed. The distribution of anthocyanin in leaves of the deep purple line “PQC” was completely concentrated in the upper epidermis, while no anthocyanin was accumulated in the mesophyll and lower epidermis, this is consistent with its phenotype as previously described. In contrast, micrographs of pakchoi leaves showed anthocyanin concentrated mainly accumulated in both the upper epidermis and lower epidermis of the lines of “PHXW”, “RWTC” and “RSH”; in particular, the anthocyanin accumulation level in the “RSH” line was much higher than that of other lines, which was attributed to the anthocyanin enriched in the upper epidermis and its adjacent mesophyll cell. A previous report revealed that the purple pigment was mainly accumulated not only in the petiole epidermis of purple turnip and purple flowering Chinese cabbage, but also in two and three leaf cell layers beneath the lower epidermis and the upper epidermis in purple Chinese cabbage [9,18]. It was suggested that differences in anthocyanin distribution might be ascribed to genetic variations, as different levels of anthocyanin distribution under the epidermal layers make purple pakchoi colorful.

### 2.3. Determination of Pigment and Nutritional Quality

**Figure 2:**
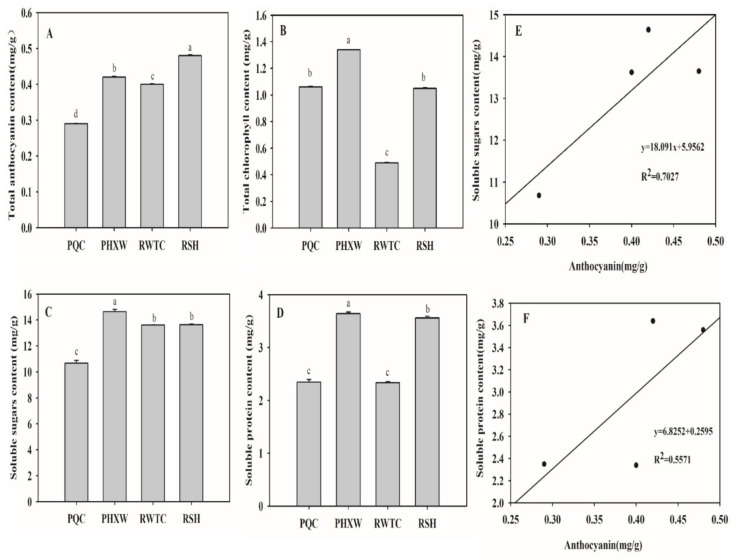
The content of pigment and nutritional quality. Content of total anthocyanin (**A**), Content of total chlorophyll (**B**), content of soluble sugar (**C**), content of soluble protein (**D**) among four purple pakchoi lines. Correlation coefficient between anthocyanin content and soluble sugar (**E**); correlation coefficient between anthocyanin content and soluble protein (**F**). Bars with different letters differ significantly (*p* < 0.05). Different lowercase letters indicate significant differences of pigment and nutritional quality among different lines (*P* < 0.05).

The anthocyanin content of the four purple pakchoi lines showed significant differences (Figure 2). The anthocyanin content in the “RSH” line was the highest (0.48 mg/g), followed by the lines of “PHXW” (0.42 mg/g) and “RWTC” (0.39 mg/g), and the “PQC” line’s anthocyanin content was the lowest (0.29 mg/g). The chlorophyll contents were higher in the “PHXW” line (1.34 mg/g), followed by the lines of “PQC” and “RSH” (1.06 mg/g, 1.05 mg/g, respectively), while the “RWTC” line was the lowest (0.49 mg/g). The ratio of anthocyanin/chlorophyll was 0.31, 0.25, 0.80 and 0.46 in the “PQC”, “PHXW”, “RWTC” and “RSH” lines, respectively. A previous study implied that the higher the proportion of anthocyanin, the redder the leaf was [22]; the chlorophyll content in the “RWTC” line was two to three times lower than those of the other lines, resulting in the highest ratio in the “RWTC” line. Therefore, the combination of anthocyanin and chlorophyll caused the leaves to be more brightly colored, leading to a leaf with a reddish purple color in the “RWTC” line. Previous research concluded that chlorophyll content had a stronger effect on purple formation [23]. With chlorophyll content increasing, the ratio of the “RSH” line was relatively lower than the ratio of ‘RWTC” line, leading to a leaf with a deep reddish purple color in the “RSH” line. However, compared with the “RSH” line, the ratios of the “PQC” line and “PHXW” line are smaller, which caused the leaves to be darker, especially in the lowest ratio of the “PHXW” line, resulting in a blackish purple leaf. In terms of nutritional quality, the contents of soluble protein and soluble sugar in the “PHXW” line were the highest, reaching 14.64 mg/g and 3.64 mg/g, respectively, followed by the lines of “RWTC” and “RSH”, while the contents of the “PQC’ line were the lowest, which showed that the new purple lines “PHXW”, “RWTC” and “RSH” were of better quality than the “PQC” line. The correlation coefficients between anthocyanin content and soluble sugar/soluble protein content were 0.70 and 0.56, respectively, showing a moderate correlation.

In this study, according to the anthocyanin/chlorophyll ratio, the color change in different purple pakchoi lines was reasonably explained. When the ratio was high, the leaf appeared to be reddish purple; when the ratio was low, the leaf appeared to be deep purple or blackish purple. Studies have shown that sugar can be used as raw material to promote the synthesis of anthocyanin, and anthocyanin content increased with the increase in sugar content [24]. This study concluded that the content of anthocyanin was positively correlated with soluble sugar and soluble protein content, which was consistent with previous research on blueberry [25] and Cotinus [5].

### 2.4. Anthocyanins Identification

**Figure 3:**
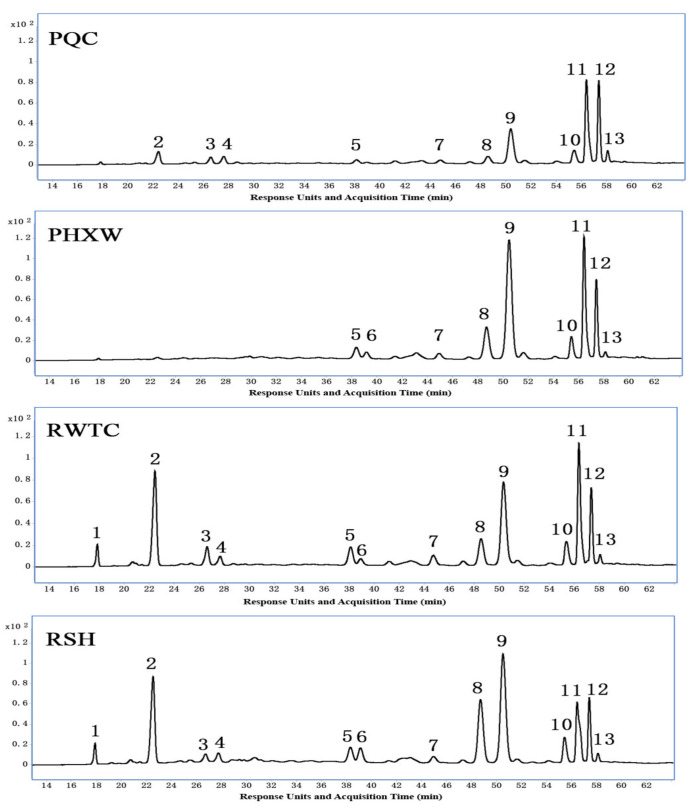
LCMS analysis profiles of anthocyanins in the four purple pakchoi lines. Samples A, B, C and D are from lines “PQC”, “PHXW”, “RWTC” and “RSH”, “PHXW”, “RWTC” and “RSH”, respectively. The peak numbers indicate the anthocyanins in Table S1.

To determine how the color formation of different purple lines was caused by anthocyanins, we detected anthocyanins in leaves of four purple pakchoi lines using the LCMS (Liquid Chromatography Mass Spectrometry) method. Thirteen anthocyanins were found in the samples, all of which existed in the lines of “RWTC” and “RSH”, and nine and 11 anthocyanins resided in “PHXW” and “PQC” lines, respectively (Figure 3). Among them, all anthocyanins had *m/z* 287 fragments, indicating that all the anthocyanins were cyanidin and cyanidin derivatives, which was completely consistent with the report by Guo et al. [9]. Acylated anthocyanins, which are acylated by aliphatic or aromatic acids (sinapic, *p-*coumaric, ferulic and caffeic), and glycosylated anthocyanins were all species that existed in the leaves of four lines. Among the cyanidin derivatives identified, peak 1 was nonacylated anthocyanin, peak 2 was monoacylated, which was acylated only by aliphatic acid, while the others were acylated by both aliphatic acid and aromatic acids. Peaks 3, 4, 5, 6, 8 and 9 were diacylated and peaks 7, 10, 11, 12 and 13 were triacylated. With regards to certain acylated anthocyanins, *cis-trans* isomers were easily produced [26,27,28]. The *cis* isomers were identified by their earlier elution times [29,30]. Two and three isomers of cyanidins (peaks 8, 9, 12), respectively, were identified simultaneously.

The relative contents of the individual anthocyanins varied in different lines; this category consisted of five primary cyanidins in purple pakchoi at relatively high levels: cyanidin 3-*trans*-(feruloyl)diglucoside-5-(malonyl)glucoside (peak 9) (Supplementary Materials Figure S1), the relative contents of which in four lines were 27.0, 42.9, 24.3 and 26.8%, respectively, followed by cyanidin 3-diglucoside-5-(malonyl)glucoside (peak 2) (Figure S2), cyanidin 3-*trans*-(*p*-coumaryl)diglucoside-5-(malonyl)glucoside (peak 8), cyanidin 3-(feruloyl)(sinapoyl)diglucoside-5-(malonyl)glucoside (peak 11) and cyanidin 3-*trans*-(*p*-coumaroyl)(sinapoyl)diglucoside-5-(malonyl)glucoside (peak 12) (Table 2). Among them, obvious differences in the relative contents of cyanidin-3-diglucoside-5-(malonyl)-glucoside (peak 2) were observed in four lines, respectively. The relative contents in the lines of “RWTC” (21.3%) and “RSH” (21.2%) were higher than those in the “PQC” line (9.5%), whereas no contents were found in the “PHXW” line. It is well known that cyanidin and peonidin are categorized as red series pigments, while delphinidin, petunidin and malvidin are classified as blue series pigments [31]; a previous study showed that the aromatic acylation of anthocyanin exhibited a shift to blue [32,33] and non-aromatic acylated cyanidin still showed the generally red color of the cyanidin [34]. By the combination of the blue and red pigments, a purple color is produced and the more acylated the aromatic group attached is, the more purple and deeply colored the leaves are. Both peak 1 and peak 2 were non-aromatic acylated cyanidin, the others were all aromatic acylated cyanidin; the ratios of non-aromatically acylated cyanidin to aromatically acylated cyanidin in the four lines were 10.4, 0, 34.9 and 34.4%, respectively (Table S1). The ratios of the “RWTC” line and “RSH” line were much higher than those of the lines of “PQC” and “PHXW”, leading to the leaves of the “RWTC” line and “RSH” line to appear reddish purple, while, with the increase in aromatically acylated cyanidin, the leaves of the “PQC” line were a deep purple. Due to the fact that cyanidin was aromatically acylated in all lines apart from the “PHXW” line, this led to a deeper purple than that of the “PQC” line, producing a more blackish purple.

The main anthocyanin components detected in deep purple lines were similar to the results reported by Zhang et al. [16], in which cyanidin 3-*trans*-(feruloyl)diglucoside-5-(malonyl)glucoside and cyanidin 3-diglucoside-5-(malonyl)glucoside accounted for 33.29 and 15.68%, respectively. However, previous studies pointed out that the main types of cyanidin 3-(sinapoyl) diglucoside-5-glucoside and cyanidin 3-(sinapoyl)(sinapoyl)diglucoside-5-glucoside, accounted for 18.3 and 17.8%, respectively [19], and cyanidin 3-(sinapoyl) diglucoside-5-(malonyl)glucoside, cyanidin 3-caffeoyl(sinapoyl) rutinoside-5-glucoside accounted for 29.1 and 22.0%, respectively [20]. The above four anthocyanins in all purple pakchoi lines were very low, and some anthocyanins were not even detected. This may be due to the fact that anthocyanin accumulation was susceptible to external environmental factors (nutritional deficiency, temperature, insolation, plant diseases and insect pests) which may not only affect the rate of anthocyanin biosynthesis, but may also exert a considerable impact on the profile of anthocyanins [35,36,37].

### 2.5. Expression Profiles of Anthocyanin Pathway Related Genes and Correlation with Total Anthocyanin Content

**Figure 4:**
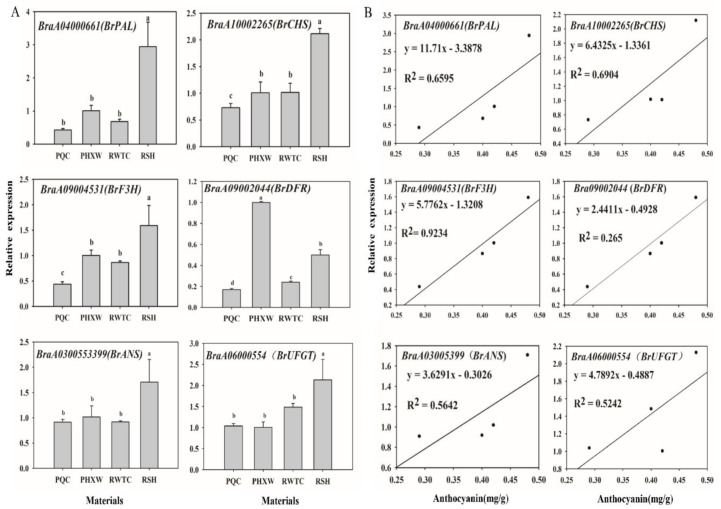
The expression of anthocyanin biosynthetic genes in four purple pakchoi lines and their correlations with anthocyanin content. The expression of six anthocyanin biosynthetic genes in four lines (**A**); correlation coefficient between anthocyanin content and the expression of anthocyanin biosynthetic genes (**B**). Different lowercase letters indicate significant differences of anthocyanin biosynthetic gene expressions among different lines (*P* < 0.05).

To clarify their potential roles in anthocyanin accumulation among four lines, the expression abundance of six anthocyanin biosynthetic genes were determined by qRT-PCR. As the results show in Figure 4A, compared with the “PQC” line, the expression levels of *BrPAL*, *BrCHS*, *BrF3H*, *BrUFGT* and *BrANS* genes were obviously up-regulated in the leaves of the “RSH” line, followed by the lines “PHXW” and “RWTC”. However, the expression levels of *BrDFR* were the highest in “PHXW”*,* followed by the lines of “RSH” and “RWTC”. To further investigate the relationship between anthocyanin accumulation and the expression levels of anthocyanin biosynthetic genes, correlation coefficients were conducted. Significant correlations were observed with early biosynthetic gene (EBG) expression—*BrPAL* (r = 0.6595), *BrCHS* (r = 0.6904) and *BrF3H* (r = 0.9234)—indicating that the expression of EBGs was consistent with the patterns of anthocyanin accumulation in four lines. However, lower correlations were observed with late biosynthetic gene (LBG) expression: *BrDFR* (r = 0.2650)*, BrANS* (r = 0.5642) and *BrUFGT* (r = 0.5242) (Figure 4B). Overall, the anthocyanin content was extremely significantly correlated with *BrF3H* gene expression. This result was in accordance with kiwifruit, which was reported the correlation between the expression level of *AaF3H* and the anthocyanin content, which was 0.98 [38]. As the central branch point of the flavonoid pathway, flavanones can be catalyzed through *F3H* to produce dihydroflavanols, which are the direct precursors of anthocyanin and flavonol synthesis. In *Antirrhinum majus* and *Petunia hybrida*, mutations at the *F3H* site can inactivate *F3H* to produce white flowers [39]. Zuker et al. reported that the antisense *F3H* gene was introduced into *Dianthus caryophyllus* and the transgenic lines partially or completely lost their original orange or red color [40]. These results indicate that *BrF3H* is a critical gene for anthocyanin biosynthesis in purple pakchoi.

## 3. Materials and Methods

### 3.1. Materials

**Figure 5:**
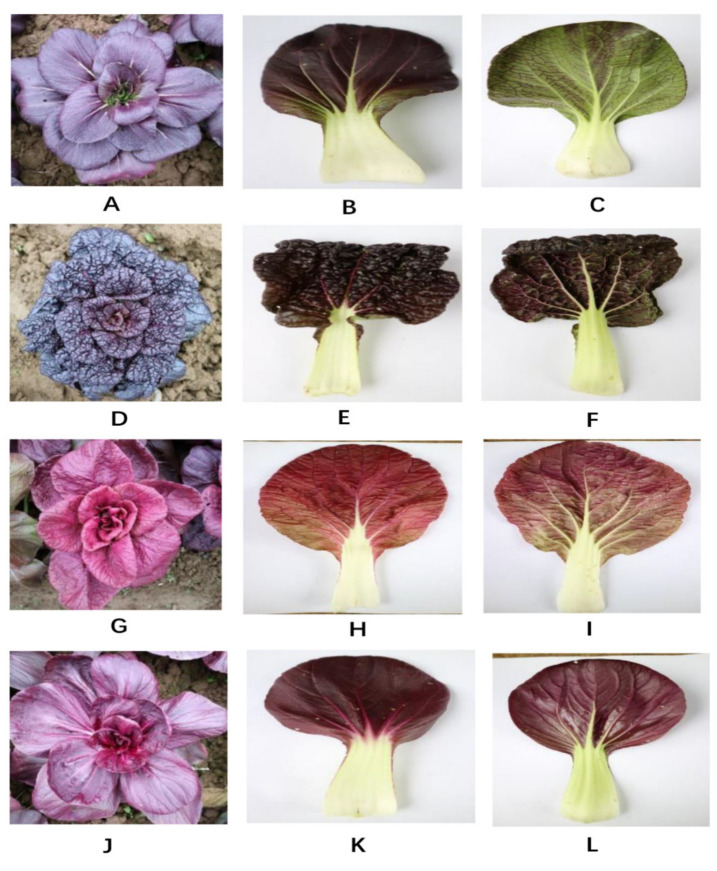
Individual plant characteristics and leaf color of four purple pakchoi lines. **A**–**C**: “PQC” line; **D**–**F**: “PHXW” line; **G**–**I**: “RWTC” line; **J**–**L**: “RSH” line; **A**, **D**, **G**, **J**: the whole plant; **B**, **E**, **H**, **K**: the front leaf surface; **C**, **F**, **I**, **L**: the back leaf surface.

Four purple pakchoi lines grown in east China (Nanjing, Jiangsu) were employed as experimental materials, showing deep purple, blackish purple, reddish purple, and deep reddish purple colors in the “PQC”, “PHXW”, “RWTC” and “RSH” lines, respectively (Figure 5). All the lines were sown on 1 September and transplanted in the field on 30 September 2018. Twenty plants were planted in each plot and each line was repeated with three plots. Forty-five days after transplanting, eight fully expanded leaves were counted from the top of the plant as samples for agronomic character observation, anatomical observation, determination of pigment and quality and LCMS analysis. Samples were frozen in liquid nitrogen and immediately stored at −80 °C.

### 3.2. Anatomical Observation of Leaf

Slices weremade for microscopic observation using free-hand sections as previously described [41]. The prepared temporary slices were quickly observed under a fluorescent microscope (Olympus CX31RTSF, Tokyo, Japan).

### 3.3. Determination of Pigment and Nutritional Quality

The chlorophyll content and anthocyanin content were measured by the ultraviolet spectrophotometry method described by Wang [42] and Zhang [43], respectively. Soluble protein content and soluble sugar content were measured by the Coomassie bright blue method and the Anthrone colorimetric method described by Wang [42], respectively.

### 3.4. Identification of Anthocyanin Components

The fresh leaves of four pakchoi (2.00 g) were ultrasonically extracted with MeOH: HCl (90:10, v) at ambient temperature, and the extract was passed through SPE C18 Sep-pak eluent to be tested. All samples were analyzed on an Agilent 1260 ultra-high-performance liquid chromatography (HPLC) system coupled with an 6530-quadrupole time-of-flight (Q-TOF) mass spectrometer operating in positive ion mode. Chromatographic separation was performed on an Agilent Zorbax SB-C18 column (4.6 × 100 mm, 1.8 μm). Separation was carried out following a 60-min multistep linear gradient using a mobile phase consisting of (A) 0.1% v aqueous formic acid in water and (B) acetonitrile from 0 to 30 min at 35% B, increased to 40% B from 30 to 45 min, then to 100% B; then, 100% B was maintained for 15 min, not including post-time. The flow rate was 0.3 mL·min^−1^; the column temperature was 45 °C; the injection volume was 5 μL; detection was performed at 530 nm. The source temperature was set at 350 °C, the capillary voltage to +4.0 kV, the N2 drying gas flow to 10 mL·min^−1^, the nebulizer pressure to 50 psi, and the fragmentor voltage to 150 V. The collision energy was set to 40 eV for MS/MS analysis. The acquired Q-TOF raw data were processed using the Agilent MassHunter B0.05.0 Workstation [44,45,46].

### 3.5. qRT-PCR Analysis

Six reported anthocyanin synthesis-related genes (*BrPAL*, *BrCHS*, *BrF3H*, *BrDFR*, *BrUFGT* and *BrANS*) [21] were chosen to analyze the expression levels in four purple pakchoi. The expression levels of the above genes were analyzed by the qRT-PCR method on the ‘LightCycler^®^ 480 II’ (Roche, Swizerland). Total RNA was extracted using Trizol Reagent according to the manufacturer’s instructions (Invitrogen, CA, USA); specific primer pairs for selected genes used in qRT-PCR were designed (Table S2). The reaction conditions were set at 95 °C for 5 min, with 40 cycles of 95 °C for 10 s, 60 °C for 30 s, and 72 °C for 15 s. The relative expression was determined using the comparative CT(Cycle Threshold) method (2^−ΔΔCT^ method) with the *actin* gene as the housekeeping gene [47].

### 3.6. Statistical Analysis

Values were expressed as means, and each value is the mean value of three biological replicates. SPSS 17.0 was employed for significance and correlation analysis. An analysis of variance (ANOVA) and Fisher’s least significant difference test (*p* ≤ 0.05) were used to compare differences among different pakchoi genotypes. Significant differences were determined at *p* ≤ 0.05.

## 4. Conclusions

In conclusion, in this study, anthocyanin distribution, profiles and expression patterns of six anthocyanin biosynthetic genes have been characterized across four purple pakchoi lines. Anthocyanin in three new purple lines was mainly concentrated in both the upper epidermis and lower epidermis; in particular, anthocyanin was enriched in the upper epidermis and its adjacent mesophyll cell, while the contents of anthocyanin, soluble sugar and soluble protein in these lines were higher than those in the deeper pakchoi line, implying good potential nutritional value. Thirteen anthocyanin components were separated and identified in four lines; all the anthocyanins were acylated and glycosylated cyanidins. The ratio of anthocyanin/chlorophyll, the ratio of non-aromatic acylated cyanidin/aromatic acylated cyanidin and the expression level of early biosynthetic genes, especially *BrF3H*, were responsible for the different purple formation of the pakchoi leaves. These results imply that new, colorful lines such as purplish red or purplish black pakchoi may be obtained by crossing lines with different anthocyanin components and anthocyanin/chlorophyll ratios. For the breeding of the purplish red line, the key is to increase the non-aromatic acylated cyanidin content and the ratio of anthocyanin/chlorophyll; for the breeding of the purplish black line, the key is to promote the content of aromatic acylated cyanidin and decrease the ratio of anthocyanin/chlorophyll, so as to achieve the requirements of breeders for the directional improvement of leaf color.

## Figures and Tables

**Table 1:** Agronomic characters of four purple pakchoi lines.

Lines	Plant Height (cm)	Plant Width (cm)	Leaf Length (cm)	Leaf Width (cm)	Petiole Length (cm)	Petiole Width (cm)	Leaf Number (piece)	Quality per Plant (g)
PQC	16.00 b	31.33 b	23.50 b	16.50 ab	7.00 c	5.90 a	25.00 ab	388.33 b
PHXW	9.33 c	26.50 c	17.17 c	10.00 c	8.17 c	2.93 d	29.67 a	160.00 c
RWTC	23.00 a	38.33 a	30.00 a	20.33 a	13.33 a	4.60 c	29.33 a	596.67 a
RSH	22.33 a	41.50 a	29.17 a	16.17 b	11.50 b	5.17 b	22.67 b	415.00 b

Note: Data are expressed as means. Different lowercase letters indicate significant differences of eight agronomic characters among different lines (*p* < 0.05).

**Table 2:** Composition of anthocyanins in purple pakchoi lines obtained by LCMS analysis.

Peak	RT (min)	M^+^ (*m/z*)	MS/MS (*m/z*)	Identification
1	17.8	773	611/449/287	Cyanidin 3-diglucoside-5-glucoside
2	22.4	859	535/287	Cyanidin 3-diglucoside-5-(malonyl)glucoside
3	26.6	1065	817/535/287	Cyanidin 3-(sinapoyl)diglucoside-5-(malonyl)glucoside
4	27.6	1035	787/535/287	Cyanidin 3-*cis*-(feruloyl)diglucoside-5-(malonyl)glucoside
5	38.1	1035	787/535/287	Cyanidin 3-*trans*-(feruloyl)diglucoside-5-(malonyl)glucoside
6	38.9	1005	757/535/287	Cyanidin 3-*cis*-(*p*-coumaryl)diglucoside-5-(malonyl)glucoside
7	44.7	1227	979/535/287	Cyanidin 3-(caffeoyl)(sinapoyl)diglucoside-5-(malonyl)glucoside
8	48.5	1005	757/535/287	Cyanidin 3-*trans*-(*p*-coumaryl)diglucoside-5-(malonyl)glucoside
9	50.3	1035	787/535/287	Cyanidin 3-*trans*-(feruloyl)diglucoside-5-(malonyl)glucoside
10	55.3	1211	963/535/287	Cyanidin 3-*cis*-(*p*-coumaroyl)(sinapoyl)diglucoside-5-(malonyl)glucoside
11	56.3	1241	993/535/287	Cyanidin 3-(feruloyl)(sinapoyl)diglucoside-5-(malonyl)glucoside
12	57.3	1211	963/535/287	Cyanidin 3-*trans*-(*p*-coumaroyl)(sinapoyl)diglucoside-5-(malonyl)glucoside
13	58.0	1181	993/535/287	Cyanidin 3-(*p*-coumaroyl)(feruloyl)diglucoside-5-(malonyl)glucoside

## Supplementary Materials

Table S1: Relative content and composition of anthocyanins in four purple pakchoi lines. Table S2: List of gene-specific primers used for qRT-PCR validation. Figure S1: Chemical structures of cyanidin 3-*trans*-(feruloyl)diglucoside-5-(malonyl)glucoside; Figure S2: Chemical structures of cyanidin 3-diglucoside-5-(malonyl)glucoside.
