# Supplementary material for: Study of the Relationship between Leaf Color Formation and Anthocyanin Metabolism among Different Purple Pakchoi Lines
Source: Molecules. 2020 Oct 19;25(20):4809. doi: 10.3390/molecules25204809 (PMC7594020; doi:10.3390/molecules25204809)
Supplement: Supplementary file 1 [file molecules-25-04809-s001.zip › Table S1 Relative content and composition of anthocyanins in four purple pakchoi lines.docx]

**Table S1** Relative content and composition of anthocyanins in four purple pakchoi lines

| Peak^a^ | Peak area of ‘PQC’ line | Ratio（%） | Peak area of  ‘PHXW’ line | Ratio  （%） | Peak area of  ‘RWTC’ line | Ratio（%） | Peak area of  ‘RSH’ line | Ratio  （%） |
| --- | --- | --- | --- | --- | --- | --- | --- | --- |
| 1 | - | 0 | - | 0 | 858006.26 | 4.6 | 822813.33 | 4.4 |
| 2 | 709287.97 | 9.5 | - | 0 | 3962314.1 | 21.3 | 4006731.1 | 21.2 |
| 3 | 245748.47 | 3.3 | - | 0 | 1120554.7 | 6 | 331347.98 | 1.8 |
| 4 | 291222.76 | 3.9 | - | 0 | 465267.76 | 2.5 | 376814.08 | 2 |
| 5 | 103135.88 | 1.4 | 515750.1 | 4 | 907431.12 | 4.9 | 580741.77 | 3.1 |
| 6 | - | 0 | 509636.98 | 4 | 737272.42 | 4 | 1123500.3 | 6 |
| 7 | 47752.72 | 0.7 | 161271.18 | 1.3 | 260786.22 | 1.4 | 93981.57 | 0.5 |
| 8 | 375429.95 | 5 | 1963828.5 | 15.2 | 1726833.4 | 9.3 | 3293190.6 | 17.4 |
| 9 | 2016244.7 | 27 | 5516488.7 | 42.9 | 4533825.3 | 24.3 | 5063974 | 26.8 |
| 10 | 320275.61 | 4.3 | 730157.87 | 5.7 | 600746.95 | 3.2 | 754318.26 | 4 |
| 11 | 1515940.3 | 20.3 | 1915499.5 | 14.9 | 1845407.4 | 9.9 | 1137171.1 | 6 |
| 12 | 1582993.8 | 21.2 | 1438446 | 11.2 | 1411288.7 | 7.6 | 1144027.3 | 6.1 |
| 13 | 253374.64 | 3.4 | 106224.64 | 0.8 | 185941.98 | 1 | 132876.09 | 0.7 |
| Total | 7461406.8 | 100 | 12857303.47 | 100 | 18615676.31 | 100 | 18861487.48 | 100 |

a. The following are the names of each anthocyanin. 1. Cyanidin 3-diglucoside-5-glucoside; 2. Cyanidin 3-diglucoside-5-(malonyl)glucoside; 3. Cyanidin 3-(sinapoyl)diglucoside-5-(malonyl)glucoside; 4. Cyanidin 3-*cis*-(feruloyl)diglucoside-5-(malonyl)glucoside; 5. Cyanidin 3-*trans*-(feruloyl)diglucoside-5-(malonyl)glucoside

6. Cyanidin 3-*cis*-(*p*-coumaryl)diglucoside-5-(malonyl)glucoside; 7. Cyanidin 3-(caffeoyl)(sinapoyl)diglucoside-5-(malonyl)glucoside; 8. Cyanidin 3-*trans*-(*p*-coumaryl)diglucoside-5-(malonyl)glucoside; 9. Cyanidin 3-*trans*-(feruloyl)diglucoside-5-(malonyl)glucoside; 10. Cyanidin 3-*cis*-(*p*-coumaroyl)(sinapoyl)diglucoside-5-(malonyl)glucoside; 11. Cyanidin 3-(feruloyl)(sinapoyl)diglucoside-5-(malonyl)glucoside; 12. Cyanidin 3-*trans*-(*p*-coumaroyl)(sinapoyl)diglucoside-5-(malonyl)glucoside; 13. Cyanidin 3-(*p*-coumaroyl)(feruloyl)diglucoside-5-(malonyl)glucoside.
