# Supplementary material for: Study of the Relationship between Leaf Color Formation and Anthocyanin Metabolism among Different Purple Pakchoi Lines
Source: Molecules. 2020 Oct 19;25(20):4809. doi: 10.3390/molecules25204809 (PMC7594020; doi:10.3390/molecules25204809)
Supplement: Supplementary file 1 [file molecules-25-04809-s001.zip › Table S2 List of gene-specific primers used for qRT-PCR validation.docx]

**Table S2** List of gene-specific primers used for qRT-PCR validation

| Target gene | Gene ID | Forward primer sequence (5' to 3') | Reverse primer sequence (5' to 3') |
| --- | --- | --- | --- |
| *BrPAL* | *BraA04000661* | AGCAACATAACCAAGATG | TCTCAGATTCTCCTCAAG |
| *BrF3H* | *BraA09004531* | ATTCATTGTCTCTAGTCATCTTC | CCGTGAGTAGTCTCTGTT |
| *BrCHS* | *BraA10002265* | TATCCTGACTACTACTTC | CTCCTTTAGAAACTCTTC |
| *BrDFR* | *BraA09002044* | CATTATGTTCAGTTCCAA | AATCTCCTTATTATCATCAC |
| *BrANS* | *BraA03005399* | TCCTGATTCCATTGTGAT | TCCTAACCTTCTCCTTATTC |
| *BrUFGT* | *BraA06000554* | GTAATGTATCCGTGGTTAG | GGTAGAGGTTAAGAGGTT |
| *Actin* |  | GTTGCTATCCAGGCTGTTC | AGCGTGAGGAAGAGCATAAC |
