# Supplementary figures and images for: Study of the Relationship between Leaf Color Formation and Anthocyanin Metabolism among Different Purple Pakchoi Lines
Source: Molecules. 2020 Oct 19;25(20):4809. doi: 10.3390/molecules25204809 (PMC7594020; doi:10.3390/molecules25204809)

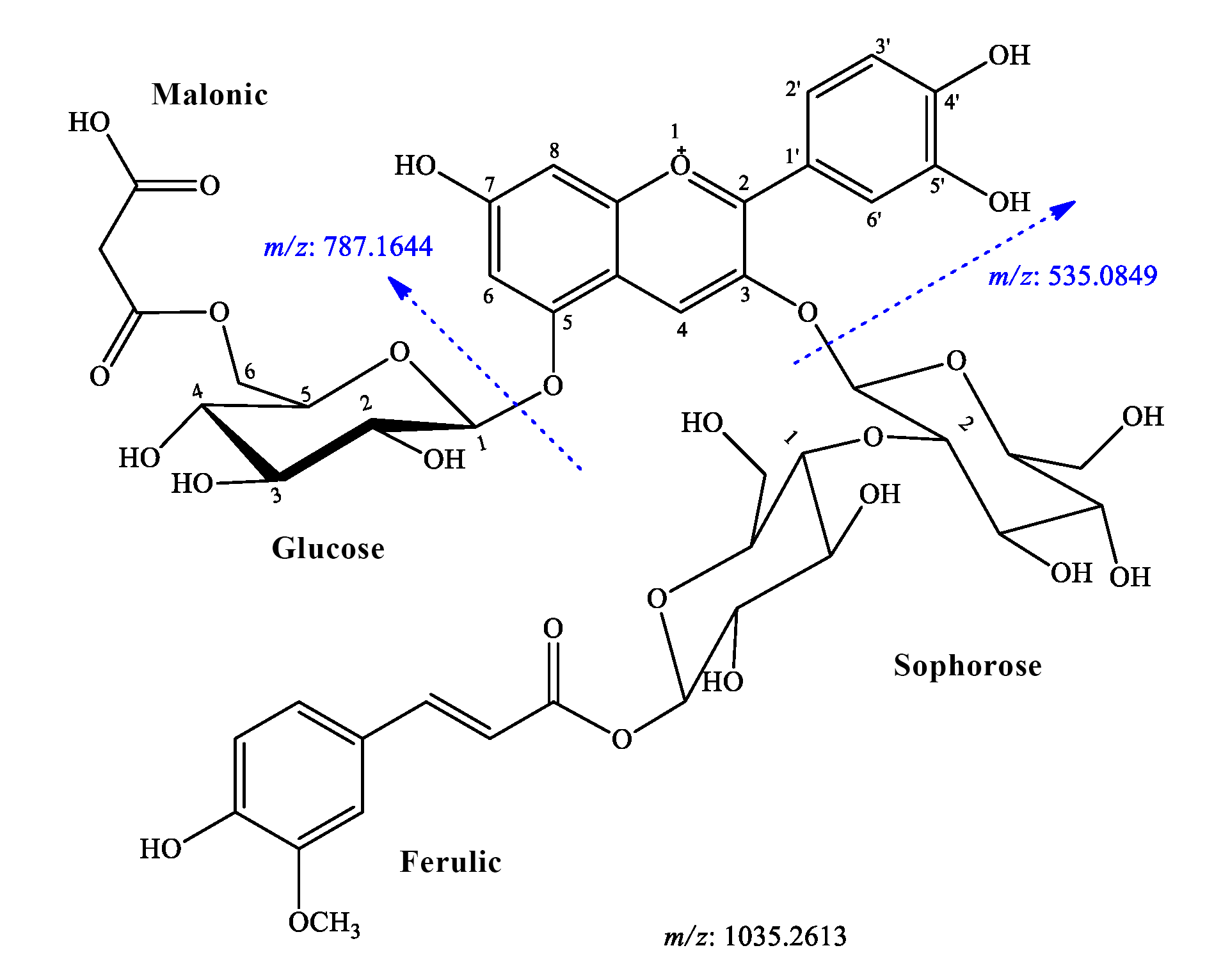


**Figure S1.** Chemical structures of cyanidin 3-*trans*-(feruloyl)diglucoside-5-(malonyl)glucoside

Supplement: Supplementary file 1 [file molecules-25-04809-s001.zip › Figure S1. Chemical structures of cyanidin 3-trans-(feruloyl)diglucoside-5-(malonyl)glucoside.docx]

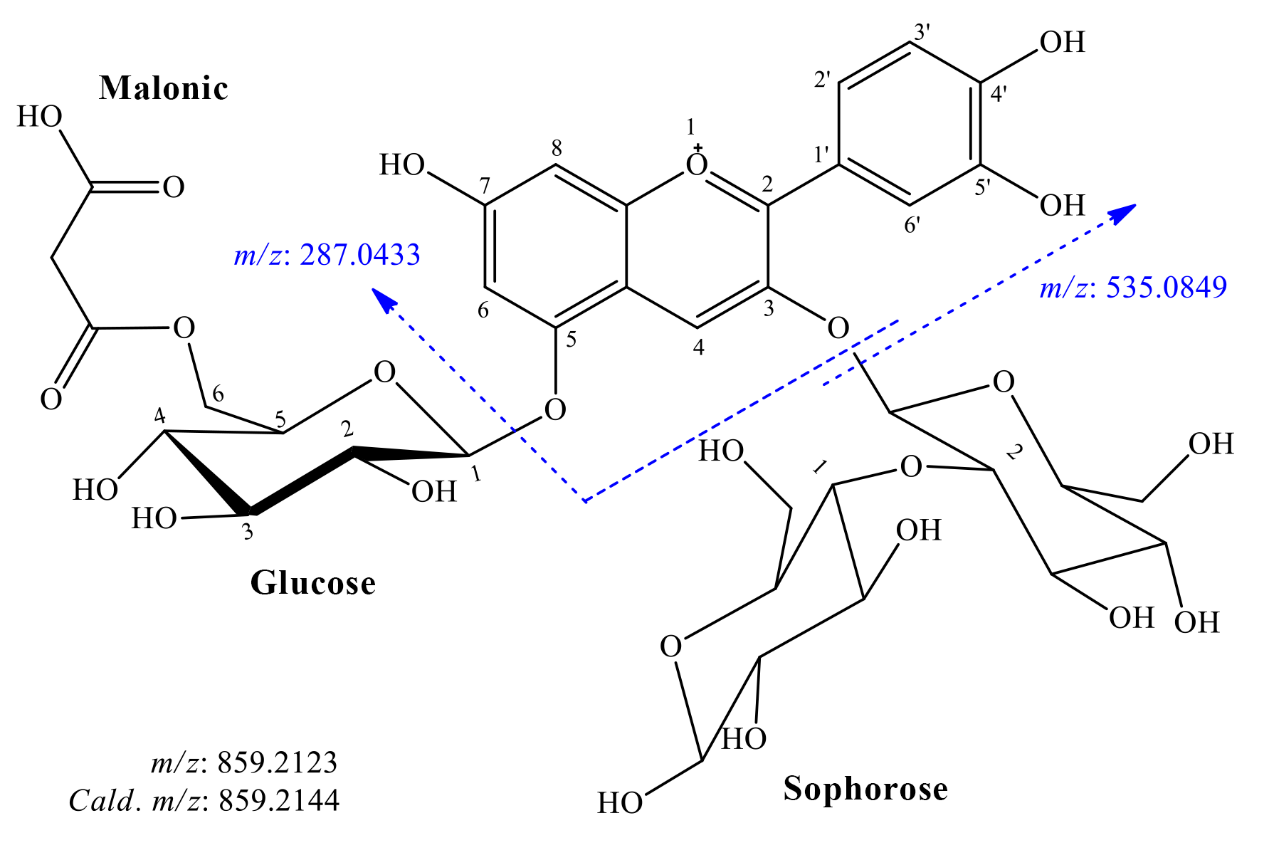


**Figure S2.** Chemical structures of Cyanidin 3-diglucoside-5-(malonyl)glucoside

Supplement: Supplementary file 1 [file molecules-25-04809-s001.zip › Figure S2. Chemical structures of Cyanidin 3-diglucoside-5-(malonyl)glucoside.docx]
